# Use of Intravesical Chemotherapy in the US Following Publication of a Randomized Clinical Trial

**DOI:** 10.1001/jamanetworkopen.2022.0602

**Published:** 2022-03-01

**Authors:** Patrick Lewicki, Spyridon P. Basourakos, Camilo Arenas-Gallo, Yuqing Qiu, Megan Prunty, Douglas S. Scherr, Jonathan E. Shoag

**Affiliations:** 1 Department of Urology, NewYork-Presbyterian Hospital, Weill Cornell Medicine, New York, New York; 2 Department of Urology, University Hospitals Cleveland Medical Center, Case Western Reserve University School of Medicine, Cleveland, Ohio; 3 Department of Healthcare Policy and Research, Weill Cornell Medicine, New York, New York

## Abstract

This cross-sectional study examines the use of intravesical chemotherapy after transurethral resection of bladder tumor before and after publication of a randomized clinical trial showing the benefits of intravesical chemotherapy.

## Introduction

Recurrence of low-grade bladder cancer is common.^1^ Intravesical chemotherapy (eg, mitomycin C or epirubicin) administered after transurethral resection of bladder tumor (TURBT) has been shown to be associated with decreased recurrence and is guideline recommended; however, rates use are low.^2,3,4^ To that end, the Southwestern Oncology Group (SWOG) S0337 randomized clinical trial (RCT), published in May 2018,^5^ sought to expand the urologist’s armamentarium by demonstrating the efficacy of post-TURBT intravesical gemcitabine. The authors of the RCT cite the drug’s lower cost, wider availability, and milder toxicity relative to mitomycin C as advantages.^5^ Whether these findings have increased rates of post-TURBT chemotherapy use is unknown. Additionally, since the trial did not compare gemcitabine directly to other historically used agents, the extent to which gemcitabine has supplanted these drugs is uncertain.

## Methods

This cross-sectional study used data from the Premier Healthcare Database, a large, all-payer sample capturing 84 994 index TURBTs between January 2015 and March 2020 via *Current Procedural Terminology* codes (52234, 52235, and 52240) across all US Census regions. Because the data source is deidentified, University Hospital Cleveland Medical Center’s institutional review board certified that this study did not constitute human participants research; thus, informed consent was not needed. This report follows the Strengthening the Reporting of Observational Studies in Epidemiology (STROBE) reporting guideline.

Billing data were queried for intravesical gemcitabine or mitomycin C; other post-TURBT intravesical chemotherapy agents were not used in this cohort. Administration of each agent was calculated by month and plotted alongside polynomial regression curves.

Variables extracted included age; hospital-reported sex, race and ethnicity, insurance, and comorbidities; and hospital academic affiliation, urbanicity, geography, and TURBT operative volume. Race and ethnicity were assessed in this study given the possible influence of socioeconomic factors on chemotherapy receipt after TURBT. A logistic regression model was used to estimate the odds of a patient receiving any post-TURBT chemotherapy (gemcitabine or mitomycin C) before and after the publication of SWOG S0337 results while adjusting for aforementioned variables. Data were analyzed from March 2021 to December 2021. A dissemination period spanning from conference abstract presentation (May 2017) to print publication of the trial (May 2018) was excluded from the comparison. Physician identification code was used to track post-TURBT chemotherapy use by surgeons before and after RCT publication. Statistical analysis was conducted using R statistical software version 4.0.3 (R Project for Statistical Computing). χ^2^ and Wilcoxon signed rank tests were used with a 2-sided significance set at α = .05.

## Results

After excluding encounters from the dissemination period, 74 259 index TURBTs were analyzed (55 409 male patients [75%]; 61 982 White patients [83%]; median [IQR] age, 72 [64-80] years) (Table). No increase was seen in the overall use of any post-TURBT chemotherapy following RCT publication, by regression analysis (odds ratio for receipt of chemotherapy after vs before publication, 0.97; 95% CI, 0.93-1.01; *P* = .11) (Figure). Patient variables associated with post-TURBT chemotherapy receipt are shown in the Table.

**Table.** Cohort Characteristics and Association With Post-TURBT Chemotherapy Receipt

Variable	Participants, No. (%)				Adjusted OR (95% CI)^b^	*P* value^c^
	Overall (N = 74 259)	Before publication (n = 41 355)	After publication (n = 32 904)	*P* value^a^		
Age, median (IQR), y	72 (64-80)	72 (64-80)	72 (64-80)	.19	1.00 (1.00-1.00)	.07
Sex						
Female	18 850 (25)	10 563 (25.5)	8287 (25.2)	.27	1 [Reference]	NA
Male	55 409 (75)	30 792 (74.5)	24 617 (74.8)		1.10 (1.05-1.15)	<.001^d^
Race or ethnicity						
White	61 982 (83)	35 034 (84.7)	26 948 (81.9)	<.001	1 [Reference]	NA
Black	4056 (5)	2188 (5.3)	1868 (5.7)		0.86 (0.78-0.94)	<.001^d^
Hispanic	2674 (4)	1266 (3.1)	1408 (4.3)		0.68 (0.61-0.77)	<.001^d^
Other^e^	5547 (7)	2867 (6.9)	2680 (8.1)		1.01 (0.94-1.09)	.71
Payer						
Self-pay	803 (1)	401 (1)	402 (1.2)	<.001	1 [Reference]	NA
Medicaid	3210 (4)	1926 (4.7)	1284 (3.9)		0.99 (0.8-1.24)	.92
Medicare	51 931 (70)	28 613 (69.2)	23 318 (70.9)		1.14 (0.93-1.4)	.22
Private	16414 (22)	9435 (22.8)	6979 (21.2)		1.29 (1.06-1.58)	.01^d^
Other	1901 (3)	980 (6.9)	921 (8.1)		1.04 (0.83-1.32)	.73
Charlson Comorbidity Index score						
≤2	50 492 (68)	30 799 (74.5)	19 693 (59.8)	<.001	1 [Reference]	NA
>2	23 764 (32)	10 553 (25.5)	13 211 (40.2)		1.16 (1.11-1.21)	<.001^d^
Hospital academic affiliation						
Academic	32 382 (44)	17 372 (42)	15 010 (45.6)	<.001	0.86 (0.83-0.9)	<.001^d^
Nonacademic	41 877 (56)	23 983 (58)	17 894 (54.4)		1 [Reference]	NA
Hospital urbanicity						
Urban	64 777 (87)	36 184 (87.5)	28 593 (86.9)	.02	0.81 (0.77-0.86)	<.001^d^
Rural	9482 (13)	5171 (12.5)	4311 (13.1)		1 [Reference]	NA
Hospital region						
Midwest	17 536 (24)	9747 (23.6)	7789 (23.7)	<.001	1 [Reference]	NA
Northeast	11 624 (16)	5964 (14.4)	5660 (17.2)		0.96 (0.9-1.03)	.28
South	32 181 (43)	17 096 (41.3)	15 085 (45.8)		1.12 (1.07-1.18)	<.001^d^
West	12 918 (17)	8548 (20.7)	4370 (13.3)		1.78 (1.68-1.89)	<.001^d^
Surgeon TURBT volume^f^						
≥75th percentile	31 652 (43)	24 241 (58.6)	18 366 (55.8)	<.001	0.92 (0.88-0.96)	<.001^d^
<75th percentile	42 607 (57)	17 114 (41.4)	14 538 (45.2)		1 [Reference]	NA
Post-TURBT chemotherapy receipt						
Yes	13 086 (18)	7431 (17.9)	5654 (17.2)	.01	NA	NA
No	61 173 (82)	33 924 (82.1)	27 250 (82.8)		NA	NA
Time period						
Before publication	NA	NA	NA	NA	1 [Reference]	NA
After publication	NA	NA	NA		0.97 (0.93-1.01)	.11

Abbreviations: NA, not applicable; OR, odds ratio; TURBT, transurethral resection of bladder tumor.

^a^ *P* values were derived from χ^2^ test comparing periods before vs after publication, except for age, which is derived from Wilcoxon signed-rank test.

^b^ Adjusted ORs were derived from a multivariable logistic regression model of characteristics associated with post-TURBT chemotherapy. The model contained all variables listed here, chosen a prioi according to suspected characteristics associated with post-TURBT chemotherapy and social determinants of health.

^c^ *P* values were derived from a multivariable logistic regression model.

^d^ These variables reached a predetermined level of significance in this model.

^e^ Other race or ethnicity was not defined in the original data set.

^f^ Surgeon TURBT volume dichotomy was based on a comparison of pseudo-*R*^2^ across multiple cutoffs.

**Figure.**
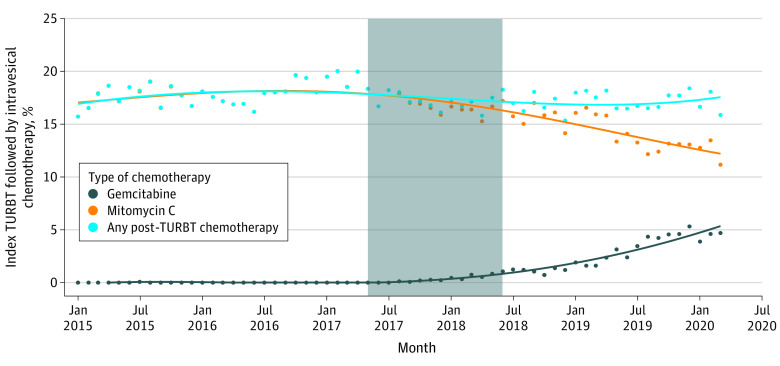
Percentage of Index Transurethral Resection of Bladder Tumors (TURBT) Procedures Followed by Intravesical Chemotherapy, by Agent Over Time Dots represent the monthly percentage for each agent, lines represent polynomial regression estimates of use, and shaded area denotes a dissemination period spanning from conference abstract presentation to print publication of trial results.

Nonetheless, use of post-TURBT gemcitabine increased following the publication of SWOG S0337, with up to 5.3% of all TURBT patients receiving the drug in March 2020 by regression estimate, compared with 0.1% before publication (percentages are regression estimates, not percentages of actual patients). By March 2020, among the 15.9% of patients (206 of 1298 patients) receiving any post-TURBT chemotherapy, 29.6% (61 of 206 patients) received gemcitabine.

Among 167 surgeons using gemcitabine who operated both before and after publication (of 6195 total over the study period), 149 (89.2%) had previously used mitomycin C, while 18 (10.8%) had not used post-TURBT chemotherapy.

## Discussion

In this cross-sectional study, we found that the overall use of any post-TURBT chemotherapy did not increase in the 22 months since the publication of the SWOG S0337 RCT that our study covered, although use of post-TURBT gemcitabine did increase. Future directions may benefit from attention to specific physician and patient factors leading to treatment omission. Indeed, evidence suggests that directed implementation strategies may improve uptake rates.^6^ Increasing gemcitabine use may, nonetheless, have systemwide benefits given its lower cost and reduced toxicity compared with mitomycin C.

Limitations of this study include the lack of clinicopathological data to adjudicate appropriate omission of postoperative chemotherapy, which is important given its indication solely for suspected low-grade disease; however, it is less parsimonious to assume that both pathology and post-TURBT chemotherapy use changed within this cohort over the study period. Freestanding ambulatory surgery centers are not captured in this data set; additionally, in-clinic instillation of chemotherapy was not assessed. A distinction cannot be made between surgeons who were unaware of the trial results vs those who may disagree with its stated conclusions.

Nonetheless, the findings of this cross-sectional study highlight the power as well as the limitations of prominent surgical RCTs. More work is needed to understand variability in adoption of new evidence.
